# The AWC ^OFF^ neuron is important for attraction to 1-butanol in *Caenorhabditis elegans*

**DOI:** 10.17912/micropub.biology.001370

**Published:** 2025-01-14

**Authors:** Vaughn E. Brown, Ella Bradley, Tymmaa A. Asaed, Sokhna B. Lo, Zach S. Bellini, Dylan J. Blackett, Jeremy J. Callaway, Jacob Hallesy, Zoey E. Joshlin, Taryn L. Kaneko, Catie H. Kaneshiro, Kae R. Kidd, Jacinda Lee, Kaitlyn M. Leung, Janelle S. Li, Ben P. Luo, Charlene C. Mbaeri, Alanna O’Neill, Precious Omomofe, James D. Schmidt, Minh Truong, Elizabeth E. Glater

**Affiliations:** 1 Molecular Biology Program, Pomona College, Claremont, California, United States of America; 2 Department of Neuroscience, Pomona College, Claremont, California, United States of America

## Abstract

*C. elegans* uses chemosensation to recognize a variety of odors, many of which are released by bacteria, the major food source of *C. elegans* . Specific amphid sensory neurons are known to detect different odorants. Here we show that the AWC ^OFF^ neuron detects the attractive odorant 1-butanol. Because few odorants that are specifically recognized by the AWC ^OFF^ neuron have been identified, we hope that the identification of this additional odorant will facilitate studies of the role of the AWC ^OFF^ neuron in odor detection and discrimination.

**Figure 1. AWC OFF neuron important for attraction of C. elegans to 1-butanol:**
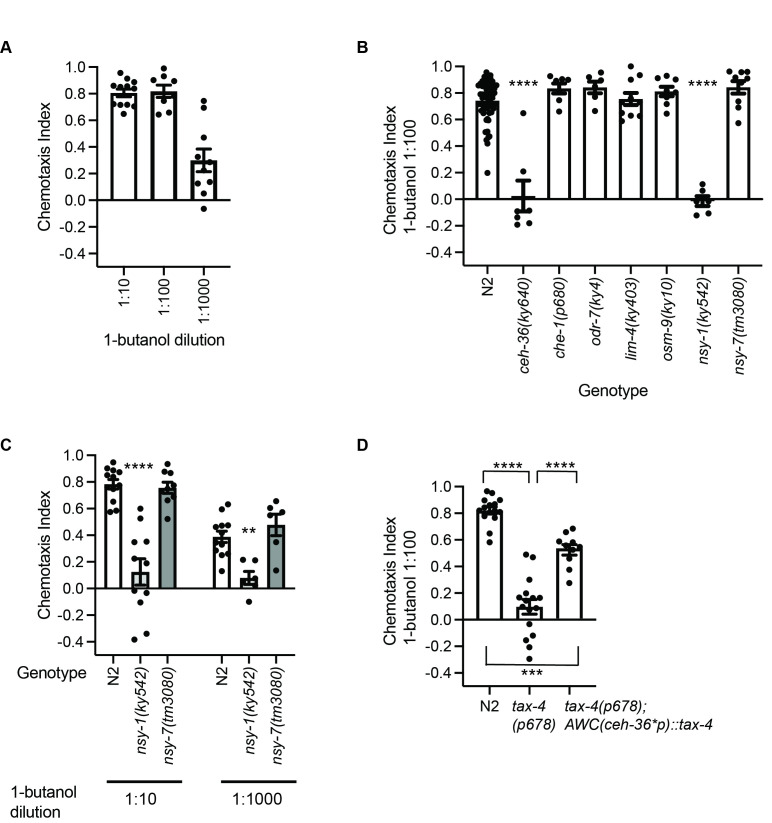
( **A** ) Wildtype N2 chemotaxis to dilutions of 1-butanol, n ≥ 8 assays. ( **B** ) 1-butanol (1:100) chemotaxis behavior of genetic mutants affecting olfactory neuron cell fates or function. Compared to N2 by ANOVA with Dunnett, n ≥ 6 assays. ( **C** ) N2, *nsy-1(ky542)* (two AWC ^ON^ neurons), and *nsy-7(tm3080)* (two AWC ^OFF^ neurons) to different dilutions of 1-butanol compared to N2 by ANOVA with Dunnett for each dilution, n ≥ 6 assays. ( **D** ) Partial rescue of 1-butanol (1:100) chemotaxis in *tax-4(p678)* mutants expressing *tax-4* cDNA in AWC (ceh-36* promoter). All genotypes compared with ANOVA with Tukey n ≥ 10 assays. For all panels, error bars are standard error of means (SEM). ****P < 0.0001, ***P < 0.001, **P < 0.01, and *P < 0.05.

## Description

*C. elegans* uses chemosensation to detect odors in its environment. Bacteria which are the major food source of *C. elegans* release many of these odors. Several chemosensory neurons located in the head or amphid of the worm detect different volatile organic chemicals and each chemosensory neuron expresses many different olfactory receptors. The primary chemosensory neurons for detecting attractive odorants are AWA and AWC and for repulsive odorants are AWB, ASH and ADL (reviewed in Ferkey et al., 2021). However, the neurons involved in detecting many odorants are still unknown. Here, we examine the neurons detecting 1-butanol. The odorant 1-butanol is found in in the headspace (volume of air above bacteria) of the following bacterial species: *E. coli* HB101 , *Flavobacterium sp.* JUb43, and *Providencia sp.* JUb39, as well as the culture media LB without bacteria (Worthy et al., 2018a).

First, we examined the chemotaxis behavior of wildtype N2 animals to 1-butanol. As previously shown (Bargmann et al., 1993) , *C. elegans* was attracted to 1-butanol at a range of dilutions, 1:10, 1:100 and 1:1000 ( Fig. 1A ). Second, to determine which neurons are involved in detecting 1-butanol, we tested *C. elegans* strains that have mutations in genes that affect the cell fate or sensory function of specific chemosensory neurons in 1-butanol chemotaxis assays. Overall, we found that mutations that affected the function or cell-fate of AWC neurons exhibited defective chemotaxis behavior to 1-butanol. The mutant *ceh-36* , which lacks AWC neurons (Lanjuin et al., 2003; Koga and Ohshima, 2004) , had defective chemotaxis to 1-butanol ( Fig. 1B ). The mutant *ceh-36* also affects ASE taste neurons (Chang et al., 2003; Koga and Ohshima 2004) , but the ASE neurons are unlikely to be involved because the *che-1* mutant, which lacks ASE neurons (Uchida et al., 2003) , exhibited wildtype chemotaxis towards 1-butanol. Other mutants affecting different chemosensory neurons exhibited wildtype 1-butanol chemotaxis. Specifically, the *odr-7* mutant which affects AWA cell fate (Sengupta et al., 1994) and *lim-4* mutant which affects AWB cell fate (Sagasti et al., 1999) resembled wildtype animals in their chemotaxis to 1-butanol. Additionally, the mutant *osm-9* which lacks sensory function of ASH, ADL and AWA neurons (Tobin et al., 2002) also showed strong chemotaxis for 1-butanol. These results suggest that AWC neurons are important for 1-butanol chemotaxis.

Next, we examined the role of AWC neurons in chemotaxis to 1-butanol. The AWC neuron class contains two neurons, AWC ^ON^ and AWC ^OFF^ , that are functionally distinct and detect different sets of overlapping odorants. AWC ^ON^ detects 2-butanone, 2-heptanone, and acetone, AWC ^OFF^ detects 2,3-pentanedione, and both detect benzaldehyde, isoamyl alcohol, and other odors (Troemel et al., 1999; Wes and Bargmann, 2001; Zhang et al., 2016; Worthy et al., 2018b; Ferkey et al., 2021) . Animals mutant for the gene *nsy-1* have cell fate transformation that results in the elimination of the AWC ^OFF^ neuron, resulting in two AWC ^ON^ neurons (Sagasti et al., 2001) . The *nsy-1* mutants were defective in 1-butanol chemotaxis indicating that AWC ^OFF^ is likely important for attraction to 1-butanol ( Fig. 1B ). Consistent with this, the *nsy-7* mutant, which lacks the AWC ^ON^ neuron and has two AWC ^OFF^ neurons (Chuang et al., 2007; Lesch et al., 2009) , retained wildtype chemotaxis for 1-butanol ( Fig. 1B ). We tested the *nsy-1* and *nsy-7* mutants at two additional dilutions (1:10 and 1:1000) of 1-butanol ( Fig. 1C ). The *nsy-1* mutant was defective at all dilutions; the *nsy-7* mutant was no different from wildtype N2 at all dilutions. Thus, having two AWC ^OFF^ neurons does not seem to increase chemotaxis to 1-butanol. Taken together, these results suggest that AWC ^OFF^ is necessary for chemotaxis to 1-butanol.

Sensory transduction in many sensory neurons, including AWC, requires a cyclic nucleotide-gated channel ( TAX-4 ) (Komatsu et al., 1996) . The *tax-4* mutant was also defective for 1-butanol chemotaxis ( Fig. 1D ). Attraction to 1-butanol was partially restored by *tax-4* expression under the ceh-36 * promoter which is selective for AWC neurons ( *ceh-36* * is a modified *ceh-36* promoter with 12 bp removed) ( Fig. 1D ) (Chang et al., 2003; Koga and Ohshima 2004) . This result provides evidence that AWC is important for chemotaxis to 1-butanol. The partial rescue may indicate that the expression level of *tax-4* in the AWC ^OFF^ neuron in the strain was not sufficient for complete rescue or that other neurons in addition to AWC are involved in chemotaxis to 1-butanol.

In conclusion, we have found that the AWC ^OFF^ is important for the detection of 1-butanol. We hope this observation will facilitate studies of AWC ^OFF^ involvement in odor detection and discrimination.

## Methods

**Chemotaxis assays**

Chemotaxis assays were performed using 10 cm square chemotaxis plates as described (Tsunozaki et al., 2008) . In brief, assay agar was 2% agar, 1mM MgSO _4_ , 1mM CaCl _2_ , 5mM phosphate buffer [pH 6.0]. Chemical dilutions were in ethanol at the concentrations indicated in figure legends. 2 μL of diluted chemical was pipetted on one side of the plate, 2 μl of ethanol on the other side, and 2 μL of 1M sodium azide on both sides to anaesthetize animals that reached odor or ethanol sources. Adult animals were washed twice in S-basal buffer and one time in water, 50–200 animals were placed at the center of chemotaxis plate, plate was covered with lid, and the distribution of animals counted after 1 hour. The *lim-4 ( ky403 )* mutant animals move more slowly and were counted after 20 hours.

**Statistical analysis**

Means represent data pooled from assays run on at least two different days with at least 6 replicates. Error bars in all figures are standard error of means. The data were analyzed using statistics described in figure legend with GraphPad Prism v10.3 for Mac (GraphPad Software, San Diego, California USA).

## Reagents

***C. elegans* strains**

**Table:** 

Strain	Genotype	Available from
N2	wildtype	CGC
CX6339	*ceh-36 ( ky640 ) *	Bargmann Lab
PR680	*che-1 ( p680 ) *	CGC
CX3937	*lim-4 ( ky403 ) *	CGC/Bargmann Lab
CX4	*odr-7 ( ky4 ) *	CGC/Bargmann Lab
CX10	*osm-9 ( ky10 ) *	CGC/Bargmann Lab
CX13078	*tax-4 ( p678 ) *	Bargmann Lab
CX9190	*nsy-1 ( ky542 ) *	Bargmann Lab
CX10232	*nsy-7 ( tm3080 ) *	Bargmann Lab
CX15111	tax-4 ( p678 ); kyEx5046 (AWC ( ceh-36 *p ^1^ ):: tax-4 sl2::GFP and *elt-2 ::GFP) *	Bargmann Lab

^1^ ceh-36*p is genomic sequence upstream of *ceh-36* gene, ctcacatccatctttctggcgactgtttca…gcctgcccccgcatgcacaa with 12bp removed, gaagaagcctta.
